# Medical and Surgical Care of Critical Burn Patients: A Comprehensive Review of Current Evidence and Practice

**DOI:** 10.7759/cureus.31550

**Published:** 2022-11-15

**Authors:** Priyankar K Datta, Sumit Roy Chowdhury, Ajisha Aravindan, Shivangi Saha, Sriharsha Rapaka

**Affiliations:** 1 Anaesthesiology, Pain Medicine and Critical Care, All India Institute of Medical Sciences, New Delhi, New Delhi, IND; 2 Neuroanaesthesiology and Critical Care, All India Institute of Medical Sciences, New Delhi, New Delhi, IND; 3 Plastic and Reconstructive Surgery, All India Institute of Medical Sciences, New Delhi, New Delhi, IND; 4 Critical Care Medicine, All India Institute of Medical Sciences, New Delhi, New Delhi, IND

**Keywords:** intensive care units, intensive care, burn, severe burns, resuscitation, inhalational injury, critical care medicine

## Abstract

Critically ill burn patients pose several unique challenges to care providers. The concepts of fluid resuscitation, nutritional management, organ support and wound care are rapidly evolving. There is a pressing need to review emerging evidence and incorporate these into practice for the effective management of burn patients. We have searched the PubMed and Google Scholar databases to review the current evidence on the acute care management of adult as well as paediatric burn patients. The rationales for current practices have been integrated into the review. The management of critically ill burn patients requires an in-depth knowledge of the pathophysiology of burn injury, a tailored approach for timely resuscitation, timely diagnosis of organ specific problems, and comprehensive wound care. This review will help the doctors and healthcare providers involved in the management of critical burn patients in their day-to-day practice.

## Introduction and background

Burn injury is defined as damage caused by heat, electricity, or chemicals to the skin and underlying tissues. Up to 22% of the burn patients attending the emergency department require ICU admission [1]. Management of such patients is challenging and requires special understanding of the multi-systemic impact of burn injuries. Although there are previously published reviews on this topic, the emergence of new evidence in recent years, particularly regarding fluid resuscitation, nutritional management, and the use of point of care ultrasound (POCUS), mandates re-visiting these topics. In this review, we discuss the comprehensive critical care management of burn patients based on current guidelines and recent evidence.

## Review

Search strategy

PubMed and Google Scholar databases were searched using the following keywords: “(burn) AND ((critical care) OR (intensive care))”; with focus on meta-analyses, randomised control trials, guidelines and review articles encompassing the intensive care management of critical burn patients from all age groups. Additionally, individual references of the review articles and meta-analyses were screened to synthesise information for this article. The Scale for the Assessment of Narrative Review Articles (SANRA) guidelines were followed while preparing this review.

Special aspects of the care of burn patients in ICU

Criteria for ICU Admission

The American Burn Association (ABA) has provided guidelines regarding which burn patients require specialised care in dedicated burn centres [2]. Of these, patients with more severe injuries require intensive supportive management, dedicated nursing care, and monitoring. Burn-care ICU admission should be considered for, but not limited to, patients with major burn injuries - adult patients with more than 20% total body surface area (TBSA) involvement (excluding first-degree burns) and more than 10% for children (<14 years) or elderly (>60 years); inhalational injury with obvious or potential airway involvement; altered mental status or shock; circumferential burns compromising respiration or limb perfusion; impending or established compartment syndrome of limbs or abdomen; evidence of evolving organ dysfunction; or associated major trauma [3].

In case of mass casualties when available resources are limited, triaging may be needed to identify and prioritise salvageable patients. The revised Baux score or the Abbreviated Burn Severity Index may be used as objective tools for this purpose [4].

Primary and Secondary Survey

Burn injuries are often associated with major trauma and the “ABCDE” sequence of Advance Burn Life Support (ABLS) protocol should be followed during initial assessment. Primary survey should start with assessment, and if needed securement, of the airway and the cervical spine. The next priority is assessment of breathing and ventilation. Acute respiratory distress in patients with burn injuries and trauma could be due to tension pneumothorax, open pneumothorax or massive haemothorax, that warrant urgent decompression. Assessment of circulatory status comes next. In case of hypotension or obvious shock, two wide-bore peripheral intravenous (IV) lines should be obtained. The burnt skin may be punctured if venous access in the areas with intact skin is not possible. Full-thickness circumferential burns may impede circulation and IV access should be avoided in that limb. In case peripheral IV cannulation is difficult, intra-osseous access or ultrasound-guided central venous access may be secured for resuscitation. Isotonic crystalloid bolus should be initiated in case patient is hypotensive. Concomitant vasopressor support may be initiated to quickly achieve desired blood pressure target. Noradrenaline is the preferred vasopressor. Once initial circulatory stability has been achieved, neurological disability should be assessed. Patients with isolated burn injuries are usually alert and conscious. Carbon monoxide (CO) or cyanide (CN) poisoning, or head injury should be suspected in obtunded patients. Full body exposure and environmental control come next. Burn patients are at increased risk of heat loss and hypothermia. Exposure should be done in a warm environment (28-33 degrees Celsius). Any adherent material, including clothes, watches, and jewelry, should be removed. For any ongoing burns or chemical burns, the involved area should be irrigated with copious amounts of water at room temperature. Cold water should never be used as it can cause hypothermia and vasoconstriction, thereby compromising dermal perfusion and increasing the depth of injury. In case of facial burns, contact lenses should be removed before oedema sets in. Patients should be examined for signs of any other major injury [5].

Following the primary survey, a detailed secondary survey should be undertaken. This includes obtaining “AMPLET” history - allergies, medications, past medical history, last meal, events leading to presentation, and tetanus immunisation history. Patients with major burns without tetanus immunisation within the last five years should be given a booster dose of toxoid. The scenario of burn injury may provide early cues regarding the patient’s status and possible complications. Patients with closed-space burns are at high risk of inhalational injury and CO and CN poisoning. Patients with high-voltage electrical burns are prone to arrhythmias, limb compartment syndrome and rhabdomyolysis. A detailed head-to-foot examination should be conducted to assess burn surface area and depth. Wallace’s rule of 9 or age-specific Lund and Browder chart may be used for burn area estimation. For scattered burns, the palm method may be used, where the surface area equivalent to the patient’s palm approximates 1% TBSA. Assessment of extremities, including peripheral pulses, is important as full thickness circumferential burns may act as a tourniquet and compromise vascular flow in the distal part of the limb. Doppler examination of arterial flow may help in early detection of such a situation, necessitating emergent intervention in the form of longitudinal escharotomy. After the detailed head-to-toe assessment, complete wound dressing and coverage should be done, followed by any radiological imaging if indicated.

Inhalational Thermal Injury

Inhalational injury should be suspected in closed space fires, history of loss of consciousness, obvious stridor or wheeze, hoarseness of voice, cough with sooty expectoration, burns involving face, oral or nasal mucosa, singeing of facial hair and nostrils, and when the patient gives history of inhalation of hot fumes.

Intubation is warranted if patient has any degree of stridor as airway oedema progresses rapidly in the first 24-48 hours with fluid resuscitation, often leading to sudden and complete airway obstruction. Extensive deep facial burns, intraoral burns, inability to swallow or clear secretions, respiratory distress, and worsening consciousness are other indications for early intubation. Paediatric patients may get fatigued quickly and supra-sternal retraction is a sign of impending respiratory failure requiring urgent intubation. Once intubated for inhalational injury, one should wait for 48-72 hours before considering extubation. Cuff leak test (CLT) should be performed prior to extubating. Extubation should be deferred if CLT is negative (i.e., if leak is absent). IV corticosteroids should be administered before attempting extubation again [6].

The medical management of inhalational injuries may include nebulised heparin - unfractionated heparin 10,000 IU every four to six hours for seven days [7]; nebulised N-acetyl cysteine (NAC) - 3ml of 20% NAC every four to six hours for seven days; nebulised bronchodilator therapy - helps in mucociliary clearance by dilating the airways [8]; and nebulised corticosteroids to reduce airway inflammation.

Flexible bronchoscopic evaluation should be performed in all intubated patients suspected to have airway inhalational injury after initial stabilisation [9]. Regular bronchoscopic suction and lavage helps clear major airways of slough and mucous plugs and reduces the chances of developing pneumonia [10]. Thermal injury to the airways is usually limited to the supraglottic region and vocal cords. Nasal flexible laryngoscopy may be performed in non-intubated patients to assess glottic oedema or inflammation in case of suspected inhalational injury. Hyperaemia and visible soot at glottic opening confirm inhalational injury. Such patients should be observed closely for signs of airway compromise.

Carbon Monoxide Toxicity

CO is produced due to incomplete oxidation of fuels because of reduced oxygen availability. The possibility of CO poisoning should be considered in all closed-space fire injuries. The affinity of CO for haemoglobin is 200-300 times that of oxygen. This results in reduced oxygen-carrying capacity of blood (anaemic hypoxia). The signs and symptoms of CO poisoning include non-specific “flu-like” symptoms - headache, dizziness, weakness, nausea, and vomiting; neurological symptoms - confusion, ataxia, stupor, and coma; cardiovascular symptoms - chest pain, ischaemic changes and arrhythmias on ECG, and cardiac failure; respiratory distress despite apparently normal oxygen saturation (SpO2) - normal pulse-oximeters cannot differentiate between carboxyhemoglobin (COHb) and oxyhemoglobin (HbO2). “Cherry-red” discolouration of skin may be seen in severe cases as a late sign.

Diagnosis of CO poisoning can be confirmed through blood gas co-oximetry. COHb level greater than 5% indicates significant CO exposure. Higher levels (5-10%) may be normally present in smokers or in persons excessively exposed to traffic fumes. Severe toxicity is defined as COHb levels >20% in adults and >15% in children.

The antidote to CO is O_2_. High-flow humidified oxygen by high-flow nasal cannula (HFNC) or non-rebreathing mask should be administered to all patients of suspected CO poisoning, irrespective of SpO2 reading, till COHb level falls below 5%. Oxygen supplementation reduces half-life of COHb from six hours to 90 minutes. Hyperbaric oxygen at 2.5-3 atmospheres pressure further reduces it to 30 minutes. If facilities are available, hyperbaric oxygen therapy should be initiated in severe cases of CO poisoning presenting with cardiovascular symptoms, ischaemic ECG changes, drowsiness or coma, COHb level >25% (>20% in pregnant patients as they are at high risk of foetal death), and in patients with severe lactic acidosis (pH <7.10) [11].

Despite optimal medical treatment, 40% of severe CO poisoning cases develop chronic neuro-cognitive impairment [12].

Cyanide Toxicity

Cyanide is produced during the process of incomplete combustion of synthetic fibres, plastics, or polymers. Clinicians should have a high index of suspicion for CN toxicity in case of closed-space fire-related smoke inhalation, and in case of industrial fires in tanning, rubber and plastic manufacturing factories. CN inhibits oxidative phosphorylation at the cellular level by binding to the ferric ion of cytochrome oxidase (histotoxic hypoxia).

The signs and symptoms of CN toxicity include neurological impairment - seizures and coma; cardiovascular collapse - unexplained shock and rising lactate; and severe abdominal pain due to bowel ischaemia. “Cherry-red” discolouration of skin may be seen. Venous blood is bright red due to high oxygen content as the tissues cannot utilise oxygen. CN poisoning may result in rapidly progressive hepatic and renal failure. It is important to note that CO and CN poisoning have almost similar presentations and are often present together.

CN toxicity can be confirmed by measuring blood CN levels. This test however is impractical as it is time-consuming and not readily available. Indirect evidence of CN toxicity includes markedly elevated blood lactate levels (>10 mmol/L) that are progressively rising despite haemodynamic support and high central venous oxygen saturation.

The management of CN poisoning requires prompt diagnosis and rapid initiation of therapy. Rescue breaths are contraindicated in suspected CN poising due to the risk of exposure of the care provider. IV hydroxocobalamin at a dose of 70mg/kg is the antidote of choice. It binds to CN and forms non-toxic cyanocobalamin which is excreted by the kidneys. The injection may be repeated once after 15 minutes in case patient does not show rapid clinical improvement. Cyanocobalamin may interfere with subsequent co-oximetry readings and causes reddish discolouration of urine. Amyl nitrite and sodium nitrite treatment are contraindicated in inhalational CN toxicity as they induce methaemoglobinemia that shifts the oxygen dissociation curve to the left and may worsen oxygen delivery to tissues in case of concomitant CO poisoning [13]. IV sodium thiosulphate (50ml 25% solution) may be used in severe toxicity not responding to hydroxocobalamin, or if hydroxocobalamin is unavailable - enzyme rhodanase converts CN to non-toxic thiocyanate using sulphur moiety from thiosulphate.

Despite timely medical treatment, patients of cyanide poisoning may develop delayed neurological sequelae including Parkinsonism [14].

Fluid Resuscitation

Skin burns are histopathologically divided into three zones: coagulation, stasis, and hyperaemia [15]. By ensuring timely perfusion of the viable stasis zone with appropriate fluid resuscitation, the increase in the coagulation zone is prevented and the skin necrosis is slowed down, thus limiting extension of the burn wound in area as well as depth. For this reason, fluid resuscitation is critical for burn patients. The goal of fluid resuscitation is to achieve and maintain euvolaemia in the face of extensive fluid losses from raw burn areas and fluid sequestration into injured tissues. These losses are most profound during the first 24 hours following thermal injury. Therefore, all patients with burn injuries involving more than 20% of TBSA should receive protocolised fluid resuscitation.

If a burn patient is hypotensive on arrival, presumably due to hypovolaemia, attempt should be made to rapidly restore intravascular volume. POCUS and dynamic indices of fluid responsiveness may help guide the initial resuscitation. Once haemodynamic stability is achieved, the maintenance phase of fluid resuscitation begins.

Balanced isotonic crystalloids are preferred for the resuscitation of burn victims. As burn patients require large volumes of resuscitation fluid, the use of normal saline may result in significant hyperchloraemic metabolic acidosis [16]. Ringer’s lactate (RL) is a balanced salt solution and does not cause metabolic acidosis. However, RL has a lower sodium concentration (130 mmol/L) than plasma, and can potentially result in the development of hyponatraemia, especially in patients with stress-induced elevated anti-diuretic hormone levels [17]. Newer balanced crystalloids have a sodium concentration closer to that of plasma and do not cause hyponatraemia.

Formal fluid resuscitation requires accurate estimation of the burn area. Prior to burn area assessment IV fluid may be stated at a rate of 10 ml/kg/h. Once burn area assessment is complete, fluid resuscitation volume should be calculated. Earlier, the Parkland formula and the modified Brooke formula were the two most widely used tools for this purpose. Using these formulae as guides, the ABA recommends calculation of resuscitation volume for the first 24 hours as 2 ml/kg/%TBSA for adults and 3 ml/kg/%TBSA for children less than 14 years of age. In addition, it is recommended that children less than 30 kg weight should also receive 5% dextrose in RL at standard maintenance rate [5].

Half of the calculated resuscitation fluid volume is given over the course of eight hours and the remaining half over the next 16 hours. These formulae only serve as a guide for resuscitation. The actual fluid rate must be titrated to individual patient needs. Urine output serves as an objective indicator of the adequacy of fluid resuscitation. The rate of fluid administration is titrated to maintain a target urine output of 0.5-1 ml/kg/h in adults and 1-1.5ml/kg/h in children. Urinary catheterisation should be considered for patients with major burns to closely monitor the adequacy of fluid resuscitation. Urine output is an indicator of volume status and organ perfusion. Diuretics should never be used during the initial resuscitation phase for the purpose of achieving urine output target. Urine output cannot be used as a guide to fluid resuscitation in patients who have received diuretics or in patients with uncontrolled blood sugar. Patients with pre-existing cardiac or renal disease should be closely monitored during fluid resuscitation. Regular POCUS examination can help guide resuscitation in such patients.

The role of colloids in resuscitation of burn patients remains controversial. The initial 24-48 hours following burn injury is a phase of extensive capillary leakage. Colloids, due to their greater vascular retentivity, can reduce the volume of resuscitation fluid required [18]. However, extensive endothelial damage caused by thermal insult may result in leakage of the colloid itself into the interstitium of injured tissues, worsening tissue oedema, delaying wound healing and potentially aggravating compartment syndrome. In addition, synthetic colloids may predispose to acute kidney injury (AKI) and coagulopathy. A meta-analysis on the use of albumin for the early resuscitation of burn patients failed to demonstrate any clear benefit in terms of mortality but showed a lower incidence of compartment syndrome in patients receiving albumin [19]. In view of the absence of any clear-cut benefit, the high cost, and the potential for serious adverse effects, many burn centres avoid the use of colloids during the first 24 hours following injury [20]. Albumin supplementation may be considered after the initial 24 hours in patients with persistent capillary leakage or extensive wound soakage, who continue to require a high rate of crystalloid infusion to maintain intra-vascular volume.

Resuscitation of patients with electrical burns is unique as the surface burn area grossly underestimates the extent of underlying tissue damage. Rhabdomyolysis is a major concern for patients with high-voltage electrical injury resulting in release of free myoglobin into plasma. Myoglobinuria initially presents with dark brown discolouration of urine. Urine dip-stick examination is positive for heme pigment. Elevated serum creatinine kinase confirms muscle injury. Maintaining adequate hydration is critical in preventing myoglobin-induced AKI. Due to the deceptively small burn surface area and importance of adequate hydration, the ABA recommends 4 ml/kg/%TBSA of resuscitation fluid over the first 24 hours for patients with electrical burns of any age, with a target urine output of 1.5-2 ml/kg/h [5]. Resuscitation with isotonic bicarbonate targeting a blood pH of 7.45-7.50 may be considered for patients with signs of myoglobinuria. Urinary alkalinisation increases the solubility of myoglobin and may prevent renal tubular injury. Hypocalcaemia is a possible complication of bicarbonate therapy and serum ionised calcium levels should be closely monitored. Mannitol, due to its antioxidant properties and tubular flushing effect, has been proposed as an agent for preventing myoglobin-induced kidney injury [21]. However, the osmotic diuresis caused by mannitol may lead to dehydration and potentially worsen kidney injury. Mannitol infusion may also result in sudden fluid shifts causing congestive cardiac failure and hypotension, and may precipitate hyperkalaemia. As such, mannitol cannot be recommended for routine use in patients with myoglobinuria.

Unless associated with major traumatic haemorrhage, blood product transfusions are rarely needed in the initial resuscitation stage of acute burns. Patients with burn injuries initially present with profound haemoconcentration due to plasma loss and haematocrit as high as 70% is often noted [22]. This haemoconcentration leads to increase in blood viscosity and further aggravates burn injury due to sluggish circulation. Blood transfusion for the purpose of volume expansion may be harmful at this stage. With early and adequate fluid resuscitation, the elevated haematocrit gradually normalises over the next 24-48 hours. Haemoglobin levels in patients with major burns may gradually fall secondary to fluid resuscitation, and due to the combined effect of acute illness, frequent blood sampling, bleeding from raw surfaces, and haemolysis due to direct thermal injury to red blood cells and vascular endothelium. Routine transfusion triggers as applicable for other critically ill patients should be used for burn victims as well.

De-resuscitation

Over-resuscitation may be as hazardous as under-resuscitation in burn patients. Excessive fluid administration may disrupt the endothelial glycocalyx leading to increased capillary permeability, further worsening the “fluid creep” into the interstitial space caused by systemic inflammatory response to the thermal injury. This increase in interstitial pressure impedes perfusion of various organs and may result in devastating consequences including acute respiratory distress syndrome (ARDS), myocardial injury and congestive heart failure, cerebral oedema, abdominal compartment syndrome (ACS), AKI, hepatic dysfunction, and compartment syndrome of limbs. Therefore, over-aggressive fluid resuscitation is to be avoided, keeping in mind that escalating fluid infusion is much easier than treating volume overload.

For the timely detection of intra-abdominal hypertension, Ivy et al. have recommended bladder pressure monitoring in patients who require more than 250 ml/kg of resuscitation fluid [23]. Beyond the initial phase of resuscitation, any patient with extensive positive fluid balance or worsening end-organ dysfunction should be assessed for fluid overload. POCUS assessment may reveal the presence of significant pulmonary oedema, pleural effusion, pericardial effusion, or ascites. Venous excess ultrasound (VExUS) assessment may be performed to look for evidence of venous congestion, especially in patients showing worsening of renal or hepatic parameters [24].

De-resuscitation (removal of excess fluid) should be considered in patients with clear evidence of fluid overload. Drainable fluid accumulation in body cavities, if associated with underlying organ dysfunction, should be tapped and drained (e.g., pleural effusion with underlying lung collapse, ascites associated with intra-abdominal hypertension, pericardial effusion with compromised cardiac filling). Fluid restriction and diuretics should be considered in patients with pulmonary oedema as evident by significant B-profile in lung ultrasound, and in patients showing evidence of severe venous congestion on VExUS scan.

Nutritional Support

Severe burn injury gives rise to a state of profound hypercatabolism mediated by catecholamines, cortisol and inflammatory cytokines. This response begins by 24-48 hours after the injury and may persist for up to two years. Consequences of this stress response include loss of muscle mass, impaired wound healing, insulin resistance and immune suppression [25]. Early initiation of nutritional support is one of the key aspects of the management of critical burn patients as it helps overcome this phase of hypercatabolism, prevents stress ulcers, reduces infections by preventing bacterial translocation from the gut, and increases immunoglobulin production. Enteral nutrition should be initiated as soon as possible (ideally within the first six to 12 hours of injury). Energy requirements in burn patients are higher than the normal resting energy expenditure (REE). Energy requirement should ideally be assessed using indirect calorimetry. However, this process is laborious and requires special equipment. In the absence of indirect calorimetry, REE can be calculated using predictive equations such as the Harris-Benedict equation or the Schofield equation and then multiplying the result by the appropriate stress factor based on TBSA involvement: 1.2 for less than 10% TBSA, 1.3 for 10-20% TBSA, 1.5 for 20-40% TBSA, 1.7 for 40-60% TBSA, and 2 for >60% TBSA [26].

The humoral stress response to severe burn not only increases the body’s energy needs but also alters how the body utilises various energy substrates. High levels of circulating catecholamines, cortisol and cytokines result in profound insulin resistance. Lipolysis and proteolysis are greatly increased after severe burn injury. Protein requirements are estimated as 1.5-2.0 g/kg/day for adults and 2-4 g/kg/day for children with major burns [27]. Increased lipolysis results in release of large amounts of free fatty acid from adipose tissue into plasma. However, the body’s ability to use free fatty acid for fuel is reduced, resulting in systemic lipotoxicity - fatty infiltration of vital organs and production of inflammatory mediators [28]. Therefore, a low-fat diet is recommended in burn patients where no more than 15-35% of total calories come from lipids [29]. A high-carbohydrate diet, in comparison to a high-fat diet, results in significantly less muscle protein degradation [27]. Guidelines recommend glutamine supplementation 0.3 g/kg/day for up to two weeks from injury [30]. Glutamine is the principal energy source for lymphocytes and enterocytes, and it may help reduce gut permeability and infective complications in burn patients. A recently published large-scale randomised control trial, however, failed to demonstrate any benefit of glutamine supplementation on mortality and length of hospitalisation among patients with moderate to severe burns [31]. Micro-nutrients that promote wound healing and require supplementation include zinc, copper, selenium, calcium, vitamin B1, C, D and E for up to one month from injury [32]. There is evidence to suggest that early high-dose IV vitamin C (1.5g every six hours) stabilises the endothelium, thereby reducing the capillary leak and the fluid resuscitation requirements by about 30% [33]. As burn injury is a state of high oxidative stress, high-dose N-acetyl cystine supplementation may be beneficial as it helps regenerate glutathione, thereby reducing oxidative free radical damage [34].

Patients with extensive burns may not be able to achieve their required nutritional intake due to various reasons - pain, sedative medications, nausea, oral mucosal involvement, facial oedema etc. In such cases Ryle’s tube feeding should be initiated early to achieve nutritional targets. Patients with severe burns often develop gastrointestinal dysmotility. In case of abdominal distension or feed intolerance, prokinetics may be initiated. Pre-emptive feeding tube insertion for nutritional support may be considered in patients with extensive burns involving >40% TBSA. Parenteral nutrition should be reserved for patients in whom enteral feed is not possible or falls grossly short of target after seven days, despite all optimising measures.

Apart from nutritional support to meet the high metabolic demand, non-nutritional management of burn-induced hyper-metabolism includes maintaining a high ambient temperature (28-30 degrees Celsius) [35], early debridement and coverage of deep burn wounds, adequate pain control, and early ambulation. Pharmacological interventions include the use of non-selective beta-blocker (propranolol) that inhibits beta-adrenergic receptor-mediated lipolysis [36], anabolic steroid (oxandrolone) [37], and, in children less than 12 months of age, recombinant human growth hormone [38].

Analgesia, Sedation and Delirium Management

Burn patients have severe pain and anxiety, and as a result are at increased risk of delirium and depression [39]. Pain assessment should be performed at least twice a day, as well as during various phases of treatment such as dressing change and ambulation. Burn-specific pain and anxiety scale (BSPAS) can be used for pain assessment [40]. Other scales frequently used include the numeric rating scale (NRS) and the visual analogue scale (VAS) in adults, and the faces pain scale in children. Critical care pain observation tool (CCPOT) can be used for non-communicative patients [41]. The nature and intensity of pain experienced by burn patients depend on the phase of injury, treatment and rehabilitation. Beyond the initial period of injury, background pain at rest is usually of a moderate intensity and can be managed with paracetamol, non-steroidal anti-inflammatory drugs (NSAIDs) and low-potency opioids. Procedural pain experienced during bedside wound dressing, debridement and physiotherapy is usually brief but of high intensity. Breakthrough pain is a spike in pain intensity when the background analgesic effect starts wearing off. This can be minimised by careful spacing of multi-modal analgesic doses. Chronic pain is pain that persists beyond healing of the skin wounds, and is usually neuropathic in nature [42].

Opioids are the mainstay of acute burn pain management. However, prolonged use of high doses of opioids may lead to tolerance, dependence, constipation, and withdrawal symptoms. The minimum effective dose to achieve desired pain control should be used. Non-opioid pharmacological measures should be used as adjuncts to opioids. Paracetamol, NSAIDs, pregabalin or gabapentin, low-dose ketamine, dexmedetomidine or clonidine, and intravenous lignocaine are useful adjunctive analgesics in those requiring high doses of opioids [43].

Burn patients undergo frequent bedside dressing and wound debridement. These procedures are extremely painful, and often turn into a major demotivating factor for patients. For adequate procedural sedation and analgesia, ketamine along with fentanyl may be used. One must ensure supplemental oxygen, monitoring of oxygen saturation and readiness for airway management during procedural sedation. Regional techniques are safe and effective, and may be considered for analgesia wherever feasible [44]. Non-pharmacological pain control therapies such as cognitive-behavioural therapy, hypnosis and distraction techniques involving immersive virtual reality are effective and should be used as adjuncts to pharmacological pain control [45].

The incidence of delirium in critically ill burn patients may be as high as 70% [46]. Delirium assessment should be done daily. Dexmedetomidine infusion may be used in haemodynamically stable patients with high anxiety levels, or evidence of delirium. For patients with hyperactive or mixed delirium, quetiapine may be used. Intravenous haloperidol may be used for acute control of agitation and aggressive behaviour in delirious patients. Benzodiazepines should be avoided as far as possible as it increases the incidence and severity of delirium.

Burn patients often complain of severe itching in the affected skin [47]. Antihistamines may be used as the first line of treatment. In case severe persistent pruritus, haloperidol and benzodiazepines may be considered. Patients with self-inflicted burn injuries or patients showing signs of clinical depression should undergo psychiatric evaluation and treatment.

Thromboprophylaxis

Burn patients are at increased risk of venous thromboembolism (VTE) with an incidence of 2.4% in patients with more than 40% TBSA burns [48]. The causes include haemoconcentration due to dehydration and capillary leak, endothelial damage due to thermal injury, prothrombotic state due to systemic inflammation, and frequently placed endovascular catheters in major veins. All bed-bound adult patients with severe burns and all paediatric burn patients on ventilator should receive VTE prophylaxis. Pharmacological prophylaxis is preferred over mechanical prophylaxis.

Low-molecular weight heparin (LMWH) is the preferred agent for pharmacological prophylaxis. Anticoagulation should be withheld in case of active bleeding from any site, significant coagulopathy, or strong suspicion of gastrointestinal haemorrhage. Mechanical thromboprophylaxis, if feasible, should be initiated in such circumstances. In case deep venous thrombosis is suspected, venous compression ultrasound should be performed to confirm the diagnosis. The affected limb should be elevated, and compression devices should be avoided. Any indwelling vascular catheter (venous or arterial) should be removed, and therapeutic anticoagulation should be initiated using twice daily dose of LMWH. Thromboprophylaxis may be discontinued once patient has started active out-of-bed mobilisation [49].

Stress Ulcer Prophylaxis

Burn patients are at increased risk of developing gastrointestinal stress ulcers (Curling’s ulcers) and should routinely be initiated on stress ulcer prophylaxis with either proton-pump inhibitors or histamine H2 receptor blockers. Patients with evidence or suspicion of gastrointestinal bleeding should additionally receive sucralfate [50]. Ulcer prophylaxis may be discontinued once resuscitation is complete and full dose oral or enteral feeding has been established [51].

Glycemic Control

Burn patients are at increased risk of developing hyperglycaemia due to exaggerated stress response. Optimal glycemic control is associated with lesser infections, better graft uptake and improved outcomes in burn patients. In comparison to standard ICU patients in whom a blood glucose target of less than 180 mg/dL appears adequate, outcomes in burn patients may be improved with tighter control in the range of 90-140 mg/dL [52].

Bowel and Bladder Management

Urine output measurement is the most widely used objective parameter for guiding fluid resuscitation in burn patients. As such, it is reasonable to catheterise patients with major burns for the first 72 hours of ICU admission to monitor hourly output. Beyond 72 hours, the further need for bladder catheterisation should be assessed. Catheter should be removed if the patient is awake, haemodynamically stable and there is no local site injury.

Patients of severe burn injury are at high risk of developing constipation due to dehydration. Patients should receive dietary soluble fibre and stool softener. In case patient has not passed stool despite laxatives for more than 72 hours, enema should be considered. Hypokalaemia and hypophosphataemia, frequently seen in burn patients because of re-feeding syndrome, may often result in paralytic ileus, and prompt correction is indicated. Diarrhoea in burn patients can be due to incomplete absorption of high feed volume causing osmotic diarrhoea, or because of broad-spectrum antibiotic treatment resulting in antibiotic-associated diarrhoea due to Clostridium difficile [53]. Osmotic diarrhoea usually resolves with reducing feed volume and protein content. Antibiotic-associated diarrhoea due to C. difficile is usually associated with other signs of infection like fever, leucocytosis, and abdominal pain. For suspected C. difficile infection, stool sample for toxin detection or culture should be sent and the patient should be initiated on oral metronidazole or oral vancomycin therapy. In case the patient progresses to paralytic ileus, intravenous metronidazole along with oral vancomycin and vancomycin enema should be administered. Faecal management systems may be considered for bed-bound patients with burns in perineal or gluteal region causing wound soilage. Some patients may require diversion colostomy [54].

Antibiotic Use and Sepsis Management

Burn wound sepsis is a common and often inevitable consequence of burn injury. Early colonisation of the burn wound within the first 24-72 hours is usually caused by Gram-positive skin commensals - Streptococcus and Staphylococcus species, from the surrounding skin. Beyond 72 hours, Gram-negative species, including Enterobacteriaceae, Pseudomonas and Acinetobacter start to translocate to the wound from the gut and from patient’s surroundings. Following initiation of Gram-negative coverage, wound colonisation is predominantly caused by Candida and filamentous fungi. Prolonged broad-spectrum antibiotic therapy may result in infection with multi-drug resistant organisms [55]. Low-grade fever of up to 102 degrees Fahrenheit is common in burn patients. It is usually a manifestation of systemic inflammation and pyrogenic cytokine release from the injured tissue. Low-grade fever without any other sign of sepsis does not necessitate antibiotic initiation or escalation. Total leucocyte count (TLC) is an unreliable indicator of sepsis in burn patients. Patients with severe burn often develop marked leukocytosis within 24 hours of the injury because of tissue injury, which is further accentuated due to haemoconcentration. This is followed by a rapid fall in TLC at about 72 to 96 hours after injury [56]. Approximately 40 to 50% of patients may develop leucopenia. Although earlier believed to be a fallout of the bone marrow suppressive effect of topical silver sulphadiazine cream, the incidence of leucopenia is similar in patients treated both with and without silver sulphadiazine [57]. The leucopenia is usually self-limiting and transient. These changes in TLC reflect the ongoing inflammatory sequence and changes in the cytokine milieu rather than infection. Serum procalcitonin (PCT) can be used as a marker for identifying bacterial sepsis in burn patients. A meta-analysis on the use of PCT for identifying sepsis in burn patients showed that a cut-off of 1.5 ng/ml had a pooled sensitivity and specificity of 0.77 and 0.65 respectively, with an area under receiver operating characteristic curve of 0.87 [58].

The use of antibiotic prophylaxis in burn patients remains controversial. A meta-analysis showed that systemic antibiotic prophylaxis in patients with severe burns may reduce the incidence of pneumonia, burn wound infection and all-cause mortality. However, an increase in the incidence of resistant infections, mainly methicillin-resistant Staphylococcus aureus (MRSA), was noted [59]. Some studies show that topical antibiotic prophylaxis with silver sulphadiazine may be associated with increased burn-wound infection and increased hospital length of stay [60]. Selective digestive tract decontamination with oral non-absorbable antibiotics has been shown to reduce the incidence of Gram-negative bacteraemia and pneumonia [61]. However, in view of low quality of evidence and ever-increasing risk of antibiotic resistance, antibiotic prophylaxis cannot be routinely recommended in patients with severe burns.

High-grade fever, rising lactate, or evidence of organ dysfunction heralds the onset of sepsis and are clear indications for initiation of broad-spectrum antibiotic cover [62]. Cultures of wound discharge, blood, urine, and endotracheal aspirate if applicable, should be sent prior to the initiation or escalation of antibiotic therapy. Source control remains the most important factor in sepsis management. Debridement of infected necrotic tissue, removal or change of indwelling catheters and vascular lines, and drainage of any collection should be considered when appropriate.

Organ Support

Respiratory and renal dysfunction are the most encountered organ failures in burn patients. Burn patients frequently require oxygen support due to ARDS or fluid overload. A trial of HFNC may be considered in tachypnoeic patients with type 1 respiratory failure. In case the clinical status worsens, intubation and invasive mechanical ventilation should be considered over a trial of non-invasive ventilation (NIV). NIV mask may be difficult to apply in patients with facial burns. Trial of NIV may unduly delay initiation of invasive mechanical ventilation resulting in crash intubations nearing cardio-respiratory arrest. Burn patients frequently have difficult airway management. Tissue injury and fluid extravasation may result in marked oedema of the face, neck, tongue, and mucosal structures. Atelectatic collapse of basal lung segments is further aggravated by mucous plugging and pleural effusion, reducing functional residual capacity. Added to this, a higher metabolic oxygen demand results in rapid desaturation during apnea. Therefore, if a patient has worsening respiratory failure, the decision to intubate should be taken early while the patient still has some respiratory reserve. Adequate preparedness is required for dealing with difficult airway situations. Rapid sequence intubation is warranted. The use of suxamethonium is contraindicated beyond 24 hours of thermal injury due to the risk of hyperkalaemia. Video laryngoscope improves success rate of intubation. Supra-glottic airway device should be kept at hand in case of difficulty in ventilation. There should be full preparedness for establishing a surgical airway if required. As with other causes of ARDS, for intubated patients, low-tidal volume lung-protective ventilation strategy should be followed. Prone ventilation may be difficult in cases with extensive burns, particularly involving the ventral aspect of the torso. Low-dose corticosteroids may be considered in patients with severe ARDS. Restrictive fluid strategy and fluid removal may benefit patients with ARDS [63].

AKI is encountered in approximately 30% of patients with severe burns [64]. The etiopathogenesis of AKI in burn patients includes prolong hypoperfusion during the initial phase of injury, rhabdomyolysis and myoglobinuria due to thermal or electrical injury to skeletal muscles, sepsis-induced, and over-resuscitation resulting in compartment syndrome. AKI contributes to mortality and increases the risk of progression of chronic kidney disease [65]. Intra-vascular volume assessment using POCUS should precede any decision regarding initiation of renal support. Hypovolaemia should be corrected. If the patient is volume overloaded, a trial of decongestion with diuretic therapy may be considered. There is no valid reason to give both fluids and diuretics to a patient. Renal replacement therapy is required in about 6-8% of burn patients and about a third of the burn patients with AKI [66].

Burn Wound Care

It is imperative for all involved care providers to have a general idea regarding the basic principles of burn wound care. Emergency resuscitation is the priority before wound care. Once the patient has been stabilised, the wound is evaluated; prior to this, the wound is covered with a sterile sheet to prevent contamination.

The goals of burn wound management are keeping the wound clean by removing the debris and contamination, facilitating removal of slough and exudates, preventing microbial growth, preventing the development of infection and sepsis, preventing damage to the viable epithelium and regenerating tissues, maintaining an environment which is conducive to wound healing, reducing pain to the injured area, and preventing the development of joint stiffness and contractures.

For practical purposes burn wounds are categorised based on the features described in Table 1.

**Table 1:** Features of partial and full thickness burns

Features	Partial thickness burns	Full thickness burns
Appearance	Pale pink with blisters	Reddish fixed-stained, leathery, black, or white
Capillary refill	Present, blanches on pressure	Absent, non-blanching
Sensation	Painful	Absent or reduced
Hair ‘pluckability’	Not easy	Easily pluckable
Healing time	2-3 weeks	Do not heal spontaneously without surgical intervention

Partial thickness burns require on average three weeks to heal on their own under conservative care, which includes applying dressings and topical antimicrobials. Full thickness burns do not heal spontaneously and usually require surgical intervention. Indeterminate burns cannot be immediately classified as partial thickness or full thickness and require a reevaluation in 10 to 14 days for formulating an appropriate surgical strategy.

**Figure 1:**
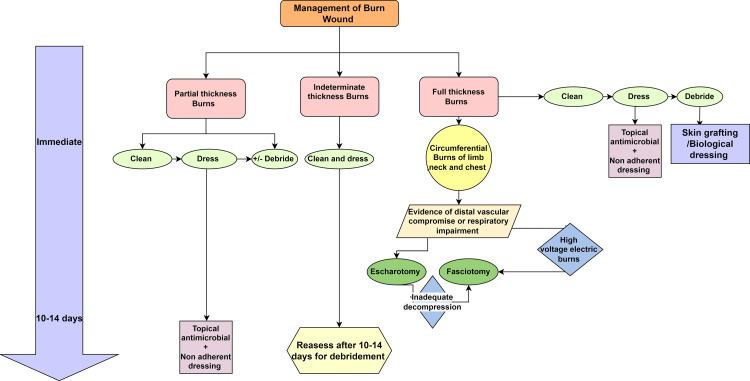
Approach to burn wound management Image credit: Shivangi Saha

The management of burn wounds has been summarised in Figure 1.

Partial thickness burn wounds are cleaned and covered with dressing materials that aid with epithelialisation. In general, antimicrobial therapy is not necessary for superficial burns, but in extensive full thickness burns, topical antimicrobials are used to prevent colonisation and to keep the area moist during the healing process. In most cases, wound care is commenced by applying a non-adherent dressing (e.g., paraffin-soaked gauze or non-adherent films) over an antibiotic ointment or cream (e.g., mupirocin or silver sulphadiazine) [67]. Depending on the type of dressing, the frequency of dressing changes might range from twice a day to once a week. The frequency of dressing changes should be sufficient to control exudate but not excessive enough to prevent wound epithelialisation. Infection is the cause of death in 75% of all burn cases after initial resuscitation. The burn wounds are initially sterile, but it is paramount to take all possible precautions to avoid colonisation and subsequent infection. Although aggressive topical wound care has been associated with reduced risk of invasive wound infections, its effectiveness has not yet been conclusively established. The various topical agents for the management of burn wounds have been compared in a Cochrane review of 56 randomized controlled trials (RCTs) with 5807 randomised participants, but most of the studies are methodologically subpar [68]. Most comparisons provided low-certainty evidence that there may be little or no difference between any of the treatments. In the absence of high-quality evidence, the decision can be made based on cost, accessibility, amount of wound exudate, frequency of dressing changes, and provider experience [69].

Full-thickness burns that are circumferential or near-circumferential can decrease skin elasticity, which when combined with ongoing resuscitation and accompanying soft tissue oedema can lead to high compartment pressures and compromised perfusion. In such cases an escharotomy is indicated. Absence of Doppler signals, reduction in the pulse volume, diminished or missing oximetry signals, elevated compartment pressures, or neurological symptoms are all indications for escharotomy. Full thickness chest burns may compromise respiration by reducing chest wall compliance and affect haemodynamics by impeding venous return [70]. In such cases, longitudinal escharotomy can be performed expeditiously at the patient’s bedside in the emergency room or the ICU. High-voltage electric burns with extensive muscle injury may develop compartment syndrome and require fasciotomy.

Eschar excision and coverage with skin graft or flap is ultimately required for full thickness burn wounds. As patients with large burn wounds may not tolerate further dermal harvesting for skin grafting (autograft), temporary dressing is frequently used before providing definitive care. If grafting is not undertaken immediately after eschar excision, mesh gauze combined with topical antimicrobial ointment or commercially available silver-containing dressings (e.g., ACTICOAT^TM^) may be used to provide a moist, minimally adherent provisional coverage that promotes healing and prevents infection. Other options for temporary wound coverage include allografts (skin graft taken from another person, living or deceased), xenografts (skin graft from non-human origin), and biologic dressings (e.g., amniotic membrane preparations). With greater availability of tissue harvesting and storage facilities, the use of deceased donor dermal allograft has increased over the years, contributing significantly towards improving outcomes in patients with extensive deep burns. Allografts form a physiological barrier against infection and provide a scaffold for re-epthelialisation of the wound, a process described as creep-substitution. Allografts are eventually rejected by the host. However, severe burn being an immunosuppressed condition, rejection is delayed, giving adequate time for the allograft to aide wound healing [71].

## Conclusions

Advances in medical management, surgical care and physiological understanding of the thermal injury process have resulted in significant improvement in the outcome of burn victims over the past decades. Yet, the short-term management and long-term rehabilitation of patients with major burn injuries remain challenging. Due to the relative paucity of large-scale clinical trials in this population, most of the practices are based on experience rather than evidence, and clinical gestalt plays a major role in the decision-making process regarding critically ill burn patients.
